# An Analysis of Four Government-Funded Reviews of Research on Homeopathic Medicine

**DOI:** 10.7759/cureus.15899

**Published:** 2021-06-24

**Authors:** Dana Ullman

**Affiliations:** 1 Family Medicine, Homeopathic Educational Services, Berkeley, USA

**Keywords:** homeopathy, nanopharmacology, complementary and alternative medicine, integrative medicine, meta-analysis, systematic reviews, health technology assessment, national health and medical research council

## Abstract

Homeopathic medicine is a controversial system of medicine that has been used worldwide for over 200 years. Recently, several governments, in part, owing to government-funded reviews of research on homeopathic medicine, have stopped reimbursements for homeopathic medicines and have discouraged their use by medical professionals. This review critically evaluates four government-funded reviews of clinical research on homeopathic medicine. An analysis of government-sponsored reviews of clinical research on homeopathic medicine was conducted, including two studies from Switzerland, one from England, and one from Australia. Three of the four government-funded reviews were critical of homeopathy, claiming that there was no reliable evidence that homeopathic medicines were effective. Three of these reviews had significant flaws, with potential ethical concerns raised in one of the reviews. The most comprehensive review of homeopathic research, including analysis of clinical and basic science concerns, found the most positive results for homeopathy.

## Introduction and background

Homeopathic medicine has been a controversial system of medicine for over 200 years. Today, homeopathy is a part of complementary and alternative medicine (CAM), as well as a component of the emerging field of nanopharmacology [1]. Around 100 million Europeans are estimated to use homeopathic medicine [2]. In 1998, homeopathy was the most frequently used CAM therapy in five out of fourteen surveyed countries in Europe and was among the three most frequently used CAM therapies in six of the remaining nine surveyed countries. Three out of four European citizens reported to be familiar with homeopathy and 29% had used homeopathic medicine for their own healthcare needs [3]. Approximately 100 million people in India depend solely on homeopathy for their healthcare needs [4].

Of significance is the considerable popularity of homeopathy among health and medical professionals in France. A large survey of licensed healthcare practitioners in France was conducted in 2011-2012 drawn from the prescribing habits from the national health insurance database [5]. A total of 6,705,420 patients were found to have had at least one reimbursement for a homeopathic medicine during a one-year period, representing over 10% of the overall population, with a higher number of females (68%) and an even higher number of children aged 0-4 years (18%). About one-third of the patients received only one reimbursement, and one-half of the patients were provided with three or more reimbursements. A total of 120,110 healthcare professionals (HCPs) in France were found to have prescribed at least one homeopathic drug, representing 43.5% of the HCPs, including 95% of general practitioners, dermatologists, and pediatricians, as well as 75% of midwives.

Despite the popularity of homeopathy in Europe, several European countries have stopped reimbursement for homeopathic treatment or homeopathic medicines. France previously reimbursed 30% of the cost of homeopathic medicine but chose to reduce it to 15% in 2020 and to eliminate it by 2021 [6]. The British National Health Services included reimbursement for homeopathic treatment by medical doctors since its inception in 1948, but in 2019, it declared “that prescribers in primary care should not initiate homeopathic items for any new patient” and should "support prescribers in deprescribing homeopathic items in all patients” [7]. In Spain, the government has stopped allowing universities to offer degrees in homeopathic medicine, announcing plans to protect the public from “pseudotherapies” such as homeopathy and acupuncture [8].

There have also been efforts to stop reimbursement for homeopathic medicines in Germany [9]; however, such efforts have not yet been successful [10]. There were even efforts to stop the support for homeopathy from the Green Party, but such efforts were also unsuccessful [11]. A proactive action for homeopathy was shown by the state government of Bavaria which chose to financially support a study researching if homeopathic medicines can help reduce the use of antibiotics for both humans and animals [12]. Subsequently, the government of Bavaria established an integrative medicine department within the Health Ministry [13].

These efforts to limit access to homeopathic medicine stem in part from recent meta-analyses and comparative reviews of homeopathic research funded by various governmental bodies that claimed no benefits from homeopathic medicines. The purpose of this article is to critically review four publications, only two of which were published in peer-reviewed journals. One of these four publications found a positive benefit from homeopathic medicines, and it is important to note that this review was the most extensive of the four studies.

## Review

An analysis of four government-sponsored reviews of clinical research on homeopathic medicine

The governments of Switzerland, Australia, and England have each funded reviews of research on the efficacy of homeopathic medicine, with each arriving at different conclusions, including two different reviews funded by the government of Switzerland. Both reviews funded by the government of Switzerland were published in peer-reviewed medical journals, while the reviews funded by the governments of Australia and England were not. An analysis of all these reviews will help provide perspective on each evaluation of scientific evidence.

The First Swiss Study

In 1998, the government of Switzerland explored the inclusion of certain CAMs, including homeopathic medicine, traditional Chinese medicine, anthroposophic medicine, herbal medicine, and neural therapy into its national health insurance system. Initially, the Swiss government provided provisional reimbursement of these alternative treatment systems while one of its governmental agencies commissioned extensive studies on these treatments to determine if they were efficacious and cost-effective.

The first study that the Swiss government funded is described below, while the second study is described next. Typical of the neutrality that Switzerland is known to embody, the Swiss government funded analyses of homeopathy from both skeptics and advocates, and predictably, each came to different conclusions.

Initially, the team of skeptics of homeopathy published their results in The Lancet [14]. This review compared 110 placebo-controlled homeopathic clinical trials with a matched group of 110 studies that tested conventional medications. The researchers then chose to compare only those clinical trials that their criteria considered to be of a similar “high quality.”

These researchers found that 21 of the homeopathic clinical trials achieved their definition of “high quality” clinical research, while only nine of the conventional clinical trials attained this standard. It would have been reasonable to assume that the researchers would then compare the “high quality” trials in both types of treatment. However, this result would have found a beneficial difference between homeopathic treatment and placebo in various ailments, and a review in a respected journal specializing in clinical research analysis confirmed this evaluation of the data [15].

Instead, this first Swiss study chose to create a smaller subset of these high-quality trials. They limited their comparative review to the largest trials in both groups to eight homeopathic trials (with at least 98 subjects) and six conventional trials (with at least 146 subjects). When reviewing only this subset of larger studies, the clinical trials were not comparable in any way, without any overlap of specific diseases. Further, the researchers claimed that one of the large high-quality trials that tested homeopathic medicines in the treatment of patients with polyarthritis did not have a comparable trial, thereby creating a reason to omit the inclusion of a large high-quality clinical study. If this trial would have been included in the meta-analysis, the authors would have had to admit a certain efficacy of homeopathy [15].

The researchers chose to place a higher value on clinical trials with larger numbers of patients because they asserted that smaller clinical trials are automatically “biased” and their results were completely ignored, even though at least seven were double-blind and placebo studies and were published in high-impact conventional medical journals, including The Lancet, BMJ, Cancer, Pediatrics Infectious Disease Journal, British Journal of Clinical Pharmacology, and Journal of Head Trauma Rehabilitation.

Further, one group of four studies on patients with various kinds of respiratory allergies, which included a total of 253 subjects and was published in BMJ [16], was not included in the final analysis without explanation. Moreover, an earlier study published in The Lancet involving 144 subjects with hay fever [17] was rated as “low quality” without explanation, despite the fact that other reviewers had rated them as “high quality” trials [18]. These clinical trials showed a significant benefit from homeopathic treatment, and their exclusion skewed the final results of this meta-analysis.

In addition to the serious methodology problems in this study, there was also either error in calculation or a direct bias. For example, in one study conducted at a Harvard hospital on brain injury, independent reviewers noted that there were three outcome criteria for the trial, two of which had positive benefits from homeopathic treatment [15]. This meta-analysis, however, only made note of the single negative outcome. 

It is also important to acknowledge that six of the eight large homeopathic trials administered the same homeopathic drug to every patient, irrespective of the different symptoms of the disease the subjects experienced. The system of homeopathy is specifically based on individualized treatment by health and medical professionals who are trained in this field. The inclusion of these six studies, therefore, may have used legally defined homeopathic medicines for patients, but the researchers did not follow the known rules in homeopathy for a proper or effective prescription. This meta-analysis even included a weight loss study in their final analysis of large high-quality trials. The study used Thyroidinum 30C (a small dose of the thyroid gland), even though this medicine is not listed in the homeopathic literature as an indicated medicine for this condition.

Perhaps one of the most contradictory statements in this meta-analysis was the authors’ acknowledgment that there were “eight trials of homeopathic remedies in acute infections of the upper respiratory tract ... indicated a substantial beneficial effect” and that this evidence is “robust.” However, the authors claim that eight studies are too few to question their conclusions, while at the same time, they based their conclusion on eight other homeopathic trials. As Fisher noted, “Is eight enough for a conclusion or not? Or does it depend on what that conclusion is” [19]?

The Second Swiss Study

The government of Switzerland funded a “health technology assessment” conducted by a group of professors from Switzerland and Germany. This group published their findings in a book [20] and in an article published in a peer-review medical journal [21]. This report on homeopathic medicine represented the most comprehensive evaluation of homeopathic medicine ever commissioned by a government. This study determined that homeopathic treatment is both effective and cost-effective and that homeopathic treatment should be reimbursed by Switzerland’s national health insurance program.

This comprehensive report systematically assessed the body of evidence from meta-analyses and from randomized double-blind and placebo-controlled clinical trials that tested homeopathic medicines. This review also reviewed observational studies of “real-world effectiveness” of homeopathic treatment as well as safety and cost-effectiveness studies. Further, the report conducted a comprehensive review of the wide body of preclinical research, including fundamental physiochemical research, in vitro studies with human cells, botanical studies, and animal trials.

This report also reviewed how the studies were conducted, both in terms of the quality of design and execution (called “internal validity”) and how appropriate each clinical trial was for the way that homeopathy is commonly practiced (called “external validity”). The subject of external validity is of special importance because some scientists and physicians conduct clinical research on homeopathy with an inadequate understanding of this system of medicine (some studies tested a homeopathic medicine that is rarely used for the condition tested, while others utilized medicines not commonly indicated for specific patients). When such studies inevitably found that the homeopathic medicine was not “effective,” it is reasonable to consider if these studies were conducted to disprove homeopathy, or if the clinical trial was an exploratory study that sought to evaluate the results of a new treatment (exploratory trials of this nature are not meant to prove or disprove the system of homeopathy but only to assess if a specific treatment for an individual with a specific condition is effective).

After evaluating the preclinical basic research as well as the high-quality clinical studies, this Swiss report claimed that homeopathic high potencies seem to induce regulatory effects (e.g., balancing or normalizing effects) and specific changes in cells or living organisms. In its evaluation of clinical research, the report determined that 20 of the 22 systematic reviews of clinical trials that tested homeopathic medicines detected at least a trend in favor of a homeopathic system of treatment.

The second Swiss report found a particularly strong body of evidence to support the homeopathic treatment of upper respiratory tract infections and respiratory allergies. Citing 29 studies in “upper respiratory tract infections/allergic reactions,” the report found 24 studies with a positive result in favor of homeopathy. Further, six out of seven controlled clinical trials that compared homeopathic treatment with conventional medical treatment found homeopathy to be more effective than conventional medical interventions (another trial found homeopathic treatment to be equivalent to conventional medical treatment). Distinct from conventional drug treatment, these results from homeopathic treatment were determined to be “free of toxic effects” from homeopathic high-potency doses (the extremely diluted medicines). When only the randomized placebo-controlled trials were reviewed, 12 out of 16 studies showed a positive result in favor of homeopathy.

After the Swiss government’s sponsorship of the above two reviews of research on homeopathic medicine, more than two-thirds of Swiss voters supported the inclusion of homeopathy and select alternative medicines in their national health insurance program in a national referendum for a test period between 2012 and 2017 [22]. Then, in 2017, the Swiss government made the determination to include homeopathic treatment as a reimbursable medical expense [23].

DS Shaw wrote a critique of this health technology assessment claiming research misconduct [24]. Shaw claimed, “almost all the authors have conflicts of interest, despite their claim that none exist.” He asserted that anyone who uses homeopathic medicine or CAM treatments has a conflict of interest, though this point of view would therefore extend to any conventional physician who would have a conflict of interest if he/she engaged in research on any conventional medical treatment. Additionally, of the nine coauthors, it should be noted that one of the authors of this Swiss report is a PhD scientist who is not engaged in any clinical treatment, two additional authors are chairpersons of their department in Medical Theory and Complementary Medicine at a major German university, and one author works for a grant-giving foundation that supports research in complementary medicine. Only one author practices homeopathic medicine.

Shaw also claimed, “the report reinterprets Kleijnen’s famous study and argues that its conclusions were only negative because the authors believed that the mechanism of homeopathy was implausible.” Shaw here is referring to a meta-analysis published in BMJ [25]. This meta-analysis reviewed 107 clinical trials, of which 81 showed that homeopathic medicines were effective, 24 showed they were ineffective, and two were inconclusive. Quoting from this article, “The amount of positive results came as a surprise to us. Based on this evidence we would be ready to accept that homeopathy can be efficacious if only the mechanism of action were more plausible.” Further, “The evidence presented in this review would probably be sufficient for establishing homeopathy as a regular treatment for certain indications.” Contrary to Shaw’s assertion, the Swiss report did not deem this meta-analysis as “negative.”

A follow-up to this 1991 meta-analysis on homeopathy that was published in The Lancet (1997) was described in detail in the Swiss report [26]. This meta-analysis concluded, “The results of our meta-analysis are not compatible with the hypothesis that the clinical effects of homeopathy are completely due to placebo.” The authors of this meta-analysis dealt directly with the issue of the implausibility of homeopathy by diminishing its importance, “Our study does, however, have major implications for future research on homeopathy. We believe that a serious effort to research homeopathy is clearly warranted despite its implausibility.”

Shaw also claimed that the authors of the Swiss report used lower-quality evidence. He claimed, “It (the report) does, however, accept evidence that would normally be excluded from a typical analysis, including observational studies, good case series and longitudinal cohort studies.” The authors of the Swiss report reviewed an extremely wide variety of evidence, including randomized double-blind and placebo-controlled trials, meta-analyses, systematic reviews, as well as “lower” quality evidence. In any case, Shaw does not present compelling evidence of research misconduct.

The Australian Report on Homeopathy

In March 2015, the Australian National Health and Medical Research Council (NHMRC) published an Information Paper on homeopathy, which is commonly referred to as “The Australian Report” [27]. This report concluded that “there are no health conditions for which there is reliable evidence that homeopathy is effective.”

The Australian Report claims that their assessment was based on an independent overview of published meta-analyses and systematic reviews, an independent evaluation of information provided by homeopathy interest groups and the public, and a review of clinical practice guidelines and government reports on homeopathy published in other countries.

There have been charges of scientific and ethical misconduct in this study. One of the more serious concerns that this report made was that it obfuscated the definition for what they meant by “reliable evidence that homeopathy is effective.” The authors of the Australian Report chose to make an arbitrary determination that studies of less than 150 subjects were “too small” to be considered “reliable.” The report asserted, “For the purposes of the homeopathy overview, studies were considered to be of sufficient size where N>150 (i.e., those studies categorized as ‘medium’ sized or larger), as the outcomes were generally continuous outcomes” [27]. However, the report did not mention these important criteria in its Executive Summary or in any of its press materials. The authors of this report did not reference a single instance that utilized this definition of “reliable” evidence in 80 similar reports issued by the Australian government. It has never been used by any other research group or agency in the world.

There are numerous clinical trials published in peer-reviewed journals that have shown that homeopathic medicines provide a therapeutic effect that is significantly different from that of a placebo, but the Australian government’s report created an additional arbitrary determination that required a study to have a minimum Jadad score of 5 (out of 5), or such evidence would be considered “unreliable.”

It should be noted that when BMJ’s “Clinical Evidence” analyzed common medical treatments to evaluate which are supported by sufficient reliable evidence, it deemed 20 subjects to be a more reasonable guideline [28]. No reference to the Jadad score was mentioned on the BMJ’s website.

Further, the Australasian Cochrane Centre wrote a critique of the Australian Report, saying, “No reliable evidence does not seem an accurate reflection of the body of evidence,” noting this was due to “A substantial proportion of small (but good quality studies) show significant differences” [29].

The private research group that wrote this report for the Australian government, Optum Insight, referenced a BMJ article for why it deemed 150 subjects to be the “minimum” necessary for any and every study to be “reliable.” In fact, this BMJ article specifically warned against using its criteria for evaluating different kinds of studies “because our results were based on meta-analyses of trials assessing binary outcomes, they cannot be extrapolated to trials assessing continuous outcomes because such trials usually differ in medical condition, risk of bias, sample size, and statistical analysis” [30]. Because a significant amount of homeopathic research is based on continuous outcomes, as acknowledged in the NHMRC Information paper itself, the authors of the Australian government’s report ignored this crucial warning.

The authors of the Australian Report also used a scientifically questionable way to evaluate results from scientific studies. On one hand, the Optum Overview Report acknowledged a significant body of scientific evidence that homeopathic medicines are effective for a wide number of acute and chronic conditions, and the report provided references to these studies verifying this efficacy. The report further confirmed that these studies were published in some of the most respected medical journals in the world, including The Lancet, BMJ, British Journal of Pharmacology, Chest (the publication of the American College of Chest Physicians), Rheumatology (the publication of the British Society for Rheumatology), Pediatrics (publication of the American Academy of Pediatrics), Cancer (journal of the American Cancer Society), European Journal of Pediatrics (publication of the Swiss Society of Pediatrics and the Belgium Society of Pediatrics), Pediatrics Infectious Disease Journal (publication of the European Society of Pediatric Infectious Diseases), and numerous others. Further, all of the above studies were randomized, double-blind, and placebo-controlled. On the other hand, for each condition evaluated in this report, the results of all trials were analyzed together as a whole, even though each trial may have tested completely different medicines and prescribing strategies, with negative trials considered to “cancel out” any positive ones.

Formal charges of scientific and ethical misconduct against the NHMRC have been filed with the Commonwealth Ombudsman. Contributing to this was the discovery of an earlier review that reported positive findings for homeopathy in some conditions but was never published. This first report was submitted in 2012 but was not accepted by the NHMRC, leading to the hiring of a new independent contractor (Optum) whose report was released in 2015. The existence of the first report was concealed from the public, but its existence was uncovered through Freedom of Information (FOI) requests.

Most recently, the Australian government’s Department of Health finally acknowledged the existence of a “first report” on homeopathy [31] after investigative efforts uncovered its existence [32]. After the release of this original report, the NHMRC acknowledged, “Contrary to some claims, the review did not conclude that homeopathy was ineffective.” Further, this original report asserted that there was “encouraging evidence” for five medical conditions: side effects of cancer therapy, otitis media, fibromyalgia, postoperative ileus, and upper respiratory tract infections. After the release of this original report, the NHMRC acknowledged in relation to the subsequent Optum review, “Contrary to some claims, the review did not conclude that homeopathy was ineffective.”

FOI requests demonstrated that Prof. Fred Mendelsohn, a member of the NHMRC’s oversight committee, confirmed the first report to be of high quality. Mendelsohn provided expert feedback to the NHMRC saying, “I am impressed by the rigor, thoroughness and systematic approach given to this evaluation [...] Overall, a lot of excellent work has gone into this review and the results are presented in a systematic, unbiased and convincing manner” [33].

Further evidence of ethical misconduct was found among members of the NHMRC committee who misrepresented themselves in terms of reporting conflicts of interest to the committee. Prof. Peter Brooks, who served as the initial committee Chair, was, in fact, a member of an anti-homeopathy lobby group but failed to disclose this conflict of interest [34].

The committee that reviewed this report for the NHMRC did not include a single expert on homeopathy, despite the NHMRC guidelines and standards mandatorily requiring the presence of such an expert.

The British Science and Technology Report

In October 2009, a House of Commons committee, called the Science and Technology Committee, created a series of colloquia with the intent to issue a report on homeopathy. The Science and Technology Committee’s report, called Evidence Check 2 [35], claimed in its mission statement that, “This inquiry was an examination of the evidence behind government policies on homeopathy, not an inquiry into homeopathy.”

This report only sought to evaluate the efficacy not the effectiveness of homeopathic medicines in clinical research under tightly controlled, artificial experimental conditions. Thus, no studies testing whether homeopathy works on “real patients” under real-world clinical conditions were allowed to be discussed or accepted as evidence.

The Science and Technology Committee initially only accepted as evidence five systematic reviews of randomized controlled trials, but after testimony from Dr. Edzard Ernst, the Committee determined that four of the five systematic reviews, which had findings in favor of homeopathy, should be excluded from their analysis. The only systematic review that this Committee deemed to be worthy was, despite its many significant flaws, the first Swiss report discussed above [14]. Ultimately, the Committee concluded that homeopathy does not work better than a placebo and that there should be no further research conducted on homeopathic medicines.

It should be noted that this Committee was composed of 14 Members of Parliament, and yet this report was approved and signed by a “majority” of only three members, with one vote against the report (the vast majority of this committee did not seem to take this investigation seriously and did not participate in any part of this Committee’s actions). Of the three votes in favor, two members were so newly appointed to this Committee that they did not attend any of the hearings.

This report was supposedly just advisory to the UK government, but the government refused to ban homeopathic products based on the recommendations of this report. Homeopathy was identified as a recognized and widely used system of medicine across the European Union, and the government further emphasized the importance of patient choice as a key reason for continuing to fund homeopathy on the NHS.

In 2017, after greater pressure from skeptics’ organizations, the NHS ended their funding for homeopathic medical services [36].

Discussion

Serious concerns on the scientific accuracy of the government-sponsored reviews of research were found in reports on homeopathy from Australia, England, and from one of the two reports from Switzerland [14].

Of special concern were the potential scientific misconduct and questionable ethical actions that surrounded the Australian report on homeopathy. The Commonwealth Ombudsman is presently investigating the NHMRC, and if they find misconduct and unethical actions, it may be appropriate for the NHMRC to retract this report, to discipline those involved in this report, to apologize to the Australian people, and to reach out and accurately educate those governments in the world that are presently seeking to evaluate what role homeopathic medicine should have in health and medical care.

Homeopathic medicine has endured over 200 years of attacks against it and misinformation about it [37,38]. Because this author’s informal assessment has determined that negative reports on homeopathy have consistently received considerably more media coverage than positive reports on homeopathy, there is a tendency for people, health and medical professionals, and state and national drug regulators to think that there is no scientific evidence that homeopathic medicines are effective.

This paper provides evidence that most of the government-sponsored meta-analyses and systematic reviews of homeopathy have had serious flaws in their analysis and conclusions. Whether these articles and reports are retracted or not, these references should be used cautiously and with a critique by observers who wish to report accurately on the state of homeopathic research today.

It may be of interest to note that the most up-to-date rigorous review of clinical research on homeopathy was published after all of these government-sponsored reviews were published. This new systematic review determined that when evaluating the highest-quality studies comparing individualized homeopathic treatment to placebo for a range of clinical conditions with what was deemed to have “reliable” evidence, the researchers found that homeopathic patients were almost twice as likely to experience a therapeutic benefit as those given a placebo (odds ratio [OR] = 1.98; 95% confidence interval [CI] = 1.16-3.38) [39]. In reviewing all 22 clinical trials identified, the patients who received homeopathic treatment experienced greater than 50% likelihood to have a therapeutic benefit than those given a placebo (OR = 1.53; 95% CI = 1.22-1.91). The researchers concluded that “Medicines prescribed in individualized homeopathy may have small, specific treatment effects.”

One of the strongest statements in this article was the confirmation that four of the five leading previous systematic reviews of homeopathic research found a benefit from homeopathic treatment over that of placebo: “Five systematic reviews have examined the RCT research literature on homeopathy as a whole, including the broad spectrum of medical conditions that have been researched and by all forms of homeopathy: four of these ‘global’ systematic reviews reached the conclusion that, with important caveats, the homeopathic intervention probably differs from placebo.”

The most significant observation of this review of homeopathic clinical research is that there is a difference between homeopathy and placebo. Some skeptics of homeopathy assert that we should not even keep an open mind about homeopathy [40], while others insist that pharmacies should not sell homeopathic drugs [41]. This review of clinical research on homeopathy suggests that the case for homeopathy is not yet closed.

## Conclusions

Three of the four government-funded reviews of research on homeopathy were critical of homeopathy, claiming that there was no reliable evidence that homeopathic medicines are effective. Three of these reviews had significant flaws, and in one case potential ethical concerns have been raised. The most comprehensive review of homeopathic research, including analysis of clinical and basic science concerns, found the most positive results for homeopathy. The most positive assessment of the efficacy of homeopathic medicines was conducted by professors and physicians who were more prone to view homeopathy favorably.

Until a systematic review is conducted by a panel of bipartisan physicians and scientists, it may be difficult to assess if homeopathic medicines are efficacious, to what degree, and for which conditions.
